# Intersecting stigma and HIV testing practices among urban refugee adolescents and youth in Kampala, Uganda: qualitative findings

**DOI:** 10.1002/jia2.25674

**Published:** 2021-03-13

**Authors:** Carmen H Logie, Moses Okumu, Daniel Kibuuka Musoke, Robert Hakiza, Simon Mwima, Peter Kyambadde, Heather Abela, Lesley Gittings, Joshua Musinguzi, Lawrence Mbuagbaw, Stefan Baral

**Affiliations:** ^1^ Factor‐Inwentash Faculty of Social Work University of Toronto Toronto Canada; ^2^ Women’s College Research Institute Women’s College Hospital Toronto Canada; ^3^ School of Social Work University of North Carolina Chapel Hill NC USA; ^4^ International Research Consortium Kampala Uganda; ^5^ Young African Refugees for Integral Development (YARID) Kampala Uganda; ^6^ National AIDS Control Program Ministry of Health Kampala Uganda; ^7^ Most At Risk Population Initiative Clinic Mulago Hospital Kampala Uganda; ^8^ Centre for Social Science Research University of Cape Town Cape Town South Africa; ^9^ Department of Health Research Methods, Evidence and Impact McMaster University Hamilton ON Canada; ^10^ Center for Public Health and Human Rights Johns Hopkins Bloomberg School of Public Health Baltimore MD USA

**Keywords:** refugee and internally displaced, HIV testing, HIV self‐testing, stigma, Uganda, adolescent and youth

## Abstract

**Introduction:**

HIV‐related risks may be exacerbated in humanitarian contexts. Uganda hosts 1.3 million refugees, of which 60% are aged under 18. There are knowledge gaps regarding HIV testing facilitators and barriers, including HIV and intersecting stigmas, among urban refugee youth. In response, we explored experiences and perspectives towards HIV testing strategies, including HIV self‐testing, with urban refugee youth in Kampala, Uganda.

**Methods:**

We implemented a qualitative study with refugee cisgender youth aged 16 to 24 living in Kampala's informal settlements from February‐April 2019. We conducted five focus groups with refugee youth, including two with adolescent boys and young men, two with adolescent girls and young women and one with female sex workers. We also conducted five key informant (KI) interviews with government, non‐government and community refugee agencies and HIV service providers. We conducted thematic analyses to understand HIV testing experiences, perspectives and recommendations.

**Results:**

Participants (n = 49) included young men (n = 17) and young women (n = 27) originally from the Democratic Republic of Congo [DRC] (n = 29), Rwanda (n = 11), Burundi (n = 3) and Sudan (n = 1), in addition to five KI (gender: n = 3 women, n = 2 men; country of origin: n = 2 Rwanda, n = 2 Uganda, n = 1 DRC). Participant narratives revealed stigma drivers included fear of HIV infection; misinformation that HIV is a “Ugandan disease”; and blame and shame for sexual activity. Stigma facilitators included legal precarity regarding sex work, same‐sex practices and immigration status, alongside healthcare mistreatment and confidentiality concerns. Stigma experiences were attributed to the social devaluation of intersecting identities (sex work, youth, refugees, sexual minorities, people living with HIV, women). Participants expressed high interest in HIV self‐testing. They recommended HIV self‐testing implementation strategies to be peer supported and expressed concerns regarding sexual‐ and gender‐based violence with partner testing.

**Conclusions:**

Intersecting stigma rooted in fear, misinformation, blame and shame, legal precarity and healthcare mistreatment constrain current HIV testing strategies with urban refugee youth. Findings align with the Health Stigma and Discrimination Framework that conceptualizes stigma drivers and facilitators that devalue intersecting health conditions and social identities. Findings can inform multi‐level strategies to foster enabling HIV testing environments with urban refugee youth, including tackling intersecting stigma and leveraging refugee youth peer support.

## 1 INTRODUCTION

Uganda has one of the world’s largest refugee populations, hosting 1.3 million forcibly displaced persons [1, 2]. Little is known about urban refugee youth’s HIV testing practices in Uganda. Although globally most refugee and displaced persons live in urban regions [3], HIV research largely focuses on the refugee settlement/camp experience [4]. The 80,000 refugees in Kampala, Uganda are often overlooked in HIV research [5]. HIV testing among this population is a critical gateway to accessing HIV prevention tools and antiretroviral therapy.

Refugee youth may have different HIV testing needs than young Ugandan nationals. Among young Ugandans aged 15 to 24, HIV prevalence is 2.1% [6]. Among young internally displaced persons in Northern Uganda aged 15 to 29, HIV prevalence was considerably higher at 12.8% [7]. Yet a study in the Uganda’s Nakivale refugee settlement reported that HIV prevalence among refugee adults was one‐quarter that of Ugandan nationals [8]. Little is known of HIV prevalence among urban refugee youth in Kampala. Youth HIV testing in Uganda falls short of the UNAIDS 95‐95‐95 targets [9], with 54% of young women and 44% of young men aged 15 to 19 ever testing [10]. HIV testing uptake may also be sub‐optimal among refugee youth in Uganda. For instance, a Kampala study reported lifetime HIV testing uptake among urban refugee young women was 54.7% and among young men was 60.7% [11].

Stigma profoundly shapes HIV testing practices [12]. A systematic review on HIV testing in countries across sub‐Saharan Africa reported that barriers included stigma and poor confidentiality in healthcare settings [13]. A qualitative systematic review reported barriers to STI and HIV services for young people in low‐ and middle‐income countries included stigma, poor confidentiality and feelings of shame in healthcare settings [14]. Refugee youth may have unique HIV testing needs. For instance refugee youth may disproportionately experience sexual‐ and gender‐based violence (SGBV), language barriers, poverty and live in contexts with overburdened health systems [15]. A qualitative study in the Nakivale settlement reported that daily priorities of food, shelter and safety needed to be addressed for adults to engage in HIV testing [16]. Systematic reviews of SRH services in humanitarian contexts have identified evidence gaps in HIV services for adolescents and youth [4, 17, 18].

Despite Uganda’s progressive legislation that allows freedom of movement, when refugees leave settlements for urban areas they receive limited social or economic assistance and largely live in informal settlements (“slums”) [2, 19]. People in informal settlements often experience overcrowding, high HIV and SGBV prevalence and poverty [20, 21, 22, 23].

HIV self‐testing is a promising strategy to reduce HIV testing barriers yet understudied among refugee youth [24]. Research in sub‐Saharan Africa with non‐refugees report that HIV self‐testing can alleviate barriers such as stigma and privacy concerns, and increase HIV testing uptake [25, 26, 27]. HIV self‐testing studies in Uganda with non‐refugee adults report it as acceptable and feasible [28, 29]. Better understanding of HIV testing practices among urban refugee youth in Uganda can inform tailored implementation strategies to improve testing coverage and linkage to care.

We address these critical knowledge gaps regarding HIV testing experiences among urban refugee youth in Kampala. Specifically, our study aims to understand a) experiences regarding HIV testing and b) HIV self‐testing perspectives, among urban refugee youth in Kampala.

## 2 METHODS

This qualitative study was conducted from February‐April 2019 with urban refugee youth, including asylum seekers, (n = 44) (aged 16 to 24) living in informal settlements in Kampala (Nsambya, Katwe, Rubaga, Kansanga, Kabalagala). We conducted five focus groups with refugee youth, including two with men, two with women and one with young female sex workers. We also conducted five key informant (KI) interviews with government, non‐government and community‐based refugee agencies and HIV providers working with refugees. Data were collected at KI workplaces or community‐based agencies in settlements.

Participants were recruited using peer‐driven sampling and convenience sampling. We trained and hired 12 peer navigators (PN) aged 18 to 24 (six women, six men) who were refugees living in the informal settlements. The interview guide was developed in collaboration with community partners and piloted with PN. PN helped to facilitate recruitment by sharing study information with peers and community collaborators shared information with their networks. Interviews and focus groups were conducted by trained local researchers, working with a translator, in English, French, Swahili, Kinyarwanda or Kirundi languages, and audio recorded. They were translated and transcribed verbatim into English.

The study was a collaboration between the community‐based refugee agencies, Ugandan researchers, Ugandan Ministry of Health and academics. Our team includes Ugandan refugee women and men and sex workers, sexually diverse persons and people with HIV in study design, implementation and analysis. We received research ethics approval from Mildmay Uganda, the Uganda National Council for Science & Technology and the University of Toronto. All participants provided informed verbal consent. In‐depth interviews were approximately 30 minutes and focus groups were approximately 45 minutes.

Our study is informed by the Health Stigma and Discrimination (HSD) Framework [12], a crosscutting framework that explores stigma processes that could reduce HIV testing uptake. Stigma is multifaceted and includes: *perceived or felt‐normative stigma*, awareness of negative community attitudes and treatment towards a social identity/health condition; *enacted stigma*, acts of discrimination and mistreatment; *anticipated stigma,* concerns that oneself will experience future prejudice; and *internalized or self‐stigma*, self‐acceptance of negative assumptions and stereotypes about one’s own health conditions/social identities [30, 31, 32]. We coded transcripts using NVIVO software, and applied thematic analyses, a theoretically flexible approach that examines inductive and deductive themes [30, 31]. Authors read and discussed transcripts, produced initial codes, generated preliminary themes by assembling codes and reviewed and defined themes [33, 34]. To enhance rigour, we applied double coding and member checking with PN. We produced a final thematic map of findings informed by the HSD Framework [12] that identifies drivers and facilitators that shape how identities, practices and/or health issues are valued and socially sanctioned [12, 33]. Stigma drivers include attitudes and beliefs that elevate HIV vulnerability, such as misinformation [35]. Facilitators are legal, institutional and community‐level influences that can enable HIV prevention *or* elevate risk. For instance enabling environments for sex workers protect rights and reduce stigma [36].

## 3 RESULTS

Focus group participants (mean age = 20.3, range 16 to 24, standard deviation = 2.2; n = 44) included men (n = 17) and women (n = 27) from the Democratic Republic of Congo (n = 29), Rwanda (n = 11), Burundi (n = 3) and Sudan (n = 1). Most (n = 42/44; 95.5%) identified as refugees, 1 as undocumented and 1 as seeking asylum. Most reported a lifetime HIV test (n = 31/44; 70.5%) and were unemployed (n = 35/44; 79.5%). KI included three women and two men (mean age = 32.8. standard deviation = 4.2; country of origin: n = 2 Rwanda, n = 2 Uganda, n = 1 DRC).

### 3.1 Stigma drivers

Narratives revealed stigma drivers included: fear of testing HIV positive; misinformation that HIV is a “Ugandan disease”; and blame and shame for sexual activity (Table 1).

**Table 1:** Illustrative quotations on stigma‐related HIV testing barriers among urban refugee adolescents and youth aged 16 to 24 in Kampala, Uganda

Theme	Sub‐theme	Illustrative quotation
Stigma drivers	Fear of testing HIV positive	Some people don't want to know their status. They want to be content with what they are. Because if she goes there and they tell her that you are [HIV] positive then she will start feeling bad. (focus group young women, 20 to 24 years, ID#6) Some of them they have that fear like ‘will I really go there and test? How will I feel if I am positive?' So, there’s that stigma with people. (key informant, refugee youth agency) You test him or her but even though he went through pretest counselling, you still see him fearing the results, he doesn't know what the outcome will be. So even the impact of the results they have not yet processed it very well because they just imagine if they find themselves positive, most of them use that statement of ‘I might kill myself ‘. They fear HIV a lot. (key informant, humanitarian agency) Once my friend discovers my [HIV] status doesn't it make a person feel bad about themselves? That is the problem I am worried about. (focus group young women, 16 to 19 years, ID#5)
	Misinformation that HIV is a “Ugandan disease”	When you tell [refugees from Country B] that we want them to come and test for HIV, they say no, no, no we don't have HIV. They say that there is no need to test. So they need a lot of sensitization. (key informant, humanitarian agency) There is a lot of stigma for example among (country A). HIV is considered a disease for Ugandans, and they do not want to test. The statistics show that very few are infected, but also very few test for HIV. Yet many of the men have more than one wife and all of these wives have never been tested. (key informant, HIV service provider)
	Blame and shame for sexual activity	Those who are negative fear going back for the next test because they think that how will the people think about them, that they go around sleeping with people, things like that, so I think most of them don't want to know. (focus group young women, 20 to 24 years, ID#6) In our culture when they see you going there for the test, they consider you as someone accusing himself that you did something wrong, that’s why you’re going for the test. And also you accuse yourself… Sometimes, although we make a mistake, instead of going to test most of them are ending up dying because of fearing the relatives or parents they’re living together with… Others think of deserting the place where they live and then say ‘Let me move to another place rather than do a test nearby to where I live’. (focus group young men, 20 to 24 years, Respondent 6)
Stigma facilitator	Legal precarity	Sex work is illegal in Uganda, and at some point you really need to be open with the health worker because you look at the fact that sometimes you will constantly go and access the medication when it comes to STDs and STIs considering the nature of the work we do, so at some point we have to disclose and say "this is the kind of work I do and that is why this and that" because the health worker would be asking why is that constantly happening? So sometimes that limits them to go to health facilities because they know that they would be asked a lot of questions and these health workers are biased. (key informant, sex work) Homosexuality is also illegal in our country, there are sex workers who sell to both men and women who are bisexuals, so that also might limit them to being comfortable accessing services. (key informant, sex work) One of our youth clients is a young man, actually after he got so sick that’s when he went for testing to find out really whether he was HIV‐positive and it turned out that he was. But, when he was having discussion with others, they were like ‘for my [refugee] case if am positive, it can go against it and I will have to keep that to myself’. Because, one of the reasons they were giving was, when I go for resettlement to a 3rd country of asylum and I tell people that I am HIV positive they will deny me a visa. (key informant, refugee youth agency)
	Healthcare mistreatment and confidentiality concerns	The stigma is double when it comes to refugees. A totally fresh [new] health worker, even telling them that you are a sex worker, even testing you for STI they take you to be filthy. Your STI test has no difference from that of a married person, but just merely knowing that you are a sex worker changes everything. Yet you tell them in case you frequently come for testing so they can understand. (key informant, sex work) Another problem which can be a barrier to access health services is poor attitudes of the health workers towards refugees specifically. For refugees there are some unique circumstances. As a government policy, refugees are allowed to access health services as Ugandans but foreigners are not. Some Ugandan health workers are not informed to differentiate between a foreigner and a refugee, and see all of them as foreigners or all as refugees. So when refugees report to us, they say that sometimes when they go to access services, that Ugandan health workers say they are tired of refugees, ‘what do you want in our country?’ So that is poor attitudes and also discrimination. (key informant, humanitarian agency) They feel like, now I am a refugee, my information will not be kept confidential at the facility, this information will be told to other people. I have heard them saying that ‘I cannot go for this testing, my information, my status will be told to other people’. (key informant, refugee youth agency) They don’t trust health workers, and they need privacy, and this is one of the reasons as to why one tests not where he/she lives. Let’s say someone lives a tent in Makindye and goes to a tent in Nsambya because people in Nsambya don’t know you. Rather than imagining that this doctor is always there and when someone else comes they can easily tell people that ‘you know this guy is HIV positive’. (focus group young men, 20 to 24 years, ID#8)

#### 3.1.1 Fear of testing HIV positive

Fear of testing and learning one’s HIV‐positive status was widespread and deterred testing, with many participants describing they would rather not know: “most people really don't want to know because if they get to know then they regret why they went to test, so they worry and grow thin and thinner day by day. They were happy before they got to know that they are positive” (focus group [FG] women, 20 to 24 years, ID#6).

Others discussed the stress produced by this fear of HIV infection: “It’s the fear of the stress if you learn that you are HIV positive so, there are many people who remain not knowing their status because they fear. Because many people say that HIV can’t kill you but the stress that comes up may kill you” (FG men, 20 to 24 years, ID#6). Fear of an HIV‐positive diagnosis was a commonly discussed strong, personal and emotional experience that interacted with and was also distinct from others’ judgements.

Participants described fear of rejection that reflects anticipated stigma from friends and others if they were to test positive and others were to find out. For instance a young man described:

Recently I went to the hospital for testing and I felt that if am positive and the society gets to know that I am positive, the way they have been treating me, it will be much different from the way they will start treating me. They will be isolating me. (focus group young men, 16 to 19 years, ID#3).

#### 3.1.2 Misinformation that HIV is a “Ugandan disease”

Key informants (KI) described perceptions among refugee communities that HIV was a “Ugandan disease” and therefore testing was not needed for refugees. For instance a KI (HIV service provider) described that this low‐risk perception may be influenced by low prevalence in a particular refugee community, yet there was also limited testing uptake. Another KI described that refugees might not believe there is HIV in their home countries:

I think we need to show them the need for testing. Now my colleagues from Country C, think there’s no HIV in their community since there’s no HIV in Country C. Even the use of condoms, many don’t use. They think they are okay! And there’s that fear about HIV. If I know that I have it, how am I going to manage? If I’m taking drugs [ART] someone will see me, and then stigma. (KI, refugee youth agency)

#### 3.1.3 Blame and shame for sexual activity

Feelings of blame and shame for being sexually active reflected the perceived stigma that youths’ sexual activity was “wrong” alongside personal concerns from engaging in sexual practices that elevated HIV exposure. These feelings were a disincentive for HIV testing. For instance a young woman described how perceived stigma towards sexually active youth deterred testing:

Some people have that fear if they put it [HIV testing service] in your community, you feel like ‘what will people say about me?’, especially when you are about fifteen, sixteen. You start asking yourself how people will think knowing that I am having sex; if not, then why am I going to do the HIV test? They have that fear. (FG women, 20 to 24 years, ID#2).

Another participant described how blaming oneself for condomless sex could reduce testing motivation:

Many are condemning themselves; seeing their past, their sexual intercourse without protecting themselves, even if there was a time the partner had to present a condom to use and never used it. So they are fearing and guess that they are HIV positive, therefore no need to go and check anymore. (focus group young men, 20 to 24 years, ID#3)

Others described this blame and shame could result in internalized stigma that may result in barriers to testing, particularly near one’s community.

### 3.2 Stigma facilitators

#### 3.2.1 Legal precarity

Stigma facilitators included legal precarity regarding sex work, same‐sex practices and immigration status. Legal precarity may exacerbate fear of anticipated stigma in healthcare settings while also contributing to internalized stigma. As a KI (sex work) described:

They are not Ugandan and some of them are not registered so that also limits them [from accessing testing] because they believe they are illegally in the country. Maybe they are also in a country where they feel they are not accepted. So there is also that self‐stigma already, because of being migrants in a country they feel they don't belong to.

Other participants described that stigma may be exacerbated among lesbian, bisexual and queer sex workers. Immigration status also emerged as a barrier to HIV testing. As a KI (refugee youth agency) described: “remember most of the refugees here, they want to go for asylum. So, if they test positive, they think there’s a possibility that they will be denied so they will not feel comfortable. The majority will not feel comfortable!”

#### 3.2.2 Healthcare mistreatment & confidentiality concerns

Participants reported enacted and anticipated stigma experiences when interacting with healthcare providers, including stigma associated with being a refugee, a young person, and/or a sex worker. For instance participants described enacted stigma in healthcare towards refugees:

There’s evidence that some medical staff were talking to refugees like animals. They don’t respect refugees, there’s intimidation: a lady reported to me that one day a staff slapped a refugee client. If you can have an officer just behave like that, how about some Ugandans who don’t know about refugees? And they have bad perceptions about refugees: they are supposed to be moody, should live in camps in villages, they shouldn’t mix, so I think it comes from this perception. (KI, refugee youth agency)

Participants also experienced enacted stigma when testing for HIV that aligns with the aforementioned perceived stigma towards sexually active youth: “Even some health workers begin blaming you for getting HIV without knowing the whole story of how you got HIV. They start asking that ‘at this young age you are HIV positive?’” (FG women, 20 to 24 years, ID#5). This was also expressed among young refugee men, who explained how healthcare workers assumed they engaged with sex workers:

When people like us youths go to the hospital for testing… the person there will start mistreating you like, ‘How come such a young kid do such and such, you are the youths who go to streets to get sex workers, you are spoilt’. So you end up losing respect in the community. That’s why, you end up like, ‘No! Even though I have sex or not, I will not go to the hospital’. (FG men, 16 to 19 years, ID#1).

Anticipated stigma was also rooted in confidentiality concerns where participants felt that healthcare workers were more likely to share HIV test results and breach confidentiality with refugees. These confidentiality concerns may result in seeking HIV testing in a different community. Others discussed mistrust due to past experiences of being tested for HIV without consent.

### 3.3 Perspectives on HIV‐self testing

Participants expressed acceptability of, and interest in, HIV self‐testing (Table 2). They recommended HIV self‐testing implementation strategies that mitigate SGBV risks and are peer supported.

**Table 2:** Illustrative quotations on HIV self‐testing perspectives among urban refugee adolescents and youth aged 16 to 24 in Kampala, Uganda

Theme	Illustrative quotation
Acceptability of HIV self‐testing	People will trust and be glad about it [HIV self‐testing]. (focus group young men, 20 to 24 years, ID#6) It’s very important for it relies on privacy, whether you are alone in your home, and you know the signs that if it shows this I am positive or negative. It can help many. (focus group young men, 20 to 24 years, ID#8) If you have explained [HIV self‐testing] to someone, they will be eager to see how it works. I mean it is a characteristic of young people to try out new things. (key informant, HIV service provider) For those that have that self‐stigma, sometimes they want to access the services but they don't even know how or they are scared to do so, this would be a very good initiative for them, that they can even test themselves from home even as they are not able to access any service. (key informant, sex work) For me I don't want to go to the hospital to get tested but if you give me that one [HIV self‐test kit], I can use it (focus group young women, 16 to 19 years, ID#8). If you give it to me today, then I can try using it tomorrow. (focus group young women, 16 to 19 years, ID#5)
Sexual‐ and gender‐based violence risks	It might also bring doubt in the family, because the woman might tell the man that let us do this self‐test and he says, don't you trust me, didn't you see the last results we got from the hospital, if I was negative then that means I am still negative. But it can even bring misunderstandings to married people like if you have your husband and he goes to work for a whole week, and he is not at home, so by the time he comes back then he says I have to test you. The husband can worry thinking the wife brought someone in the house. (focus group young women, 20 to 24 years, ID#4) It may create some gender violence. Trust me when someone brings it home, the partner will want to see those results come what may. Just imagine if that result is positive, automatically you will be identified as a womanizer and vice versa. Let me give an example. Among the (x nationality), if a woman turns positive and the man is negative, the woman will be considered wasted because she has acquired ‘bad manners’ of Ugandans. (key informant, HIV service provider) If he brings it [HIV self‐testing kit], she may think that I don’t trust her, and if she’s the one who brought it to me, still I would think the same way. That will show that there’s no trust. (focus group young men, 20 to 24 years, ID#4)
Peer‐supported HIV self‐testing	The truth is that it is hard for the sex workers… So most times we feel more comfortable with the sex worker peer talking to me because I know that this person is not judging me, this person is not discriminating me, this person understands me. (key informant, sex work) I prefer the peer educator because they are more experienced at that, because after testing I could find myself positive so they can comfort me and they cannot tell anyone even when I am HIV positive. But for a friend they can give it to me but after they come asking what the results were, they will insist and that can bring about stress. If you refuse to tell they may think you have HIV. (focus group young women, 20 to 24 years, ID#9) If it was in the facility and there was an assistant who may be a fellow refugee, they would feel comfortable to do the test. But, if it not the case, maybe they might not feel comfortable simply because, they don’t feel like one of them is there. Remember in the refugee community they understand and trust each other very well. (key informant, refugee youth agency) Peer educators from health centres, or who have a background on medical issues, it would be better if those peer educators are part of health centres so that we can trust them. (focus group young women, 16 to 19 years, ID#4) A peer educator because he is a leader. He gets a lot of information about health and a lot of trainings and as leader shares information with his people. (focus group young men, 20 to 24 years, ID#4). A peer educator is someone you trust, who has counselled, advised you in many areas. So, when he brings this kind of kit, you will still trust him. (focus group young men, 20 to 24 years, ID#3)

#### 3.3.1 Acceptability of HIV self‐testing

These experiences of intersecting stigmas contributed to a strong interest in HIV self‐testing as participants felt this was a more confidential HIV testing approach. As put by one participant: “Everyone would be interested in using (a self‐test) because this one you know that you are going to test yourself and you are the one to know those results. Even the mother can't know that you have tested yourself or that you have HIV. So that one would be the best option to use among the youth” (FG women, 20 to 24 years, ID#6).

HIV self‐testing was also perceived as a strategy that could reduce internalized stigma:

They would be much willing (to take a self‐test) considering the challenges we have had when it comes to self‐stigma and all that, this is something that totally addresses that need because we didn't have any other option apart from either pushing them to health facilities or conducting community self‐testing. This would be welcome. (KI, sex work)

#### 3.3.2 Sexual‐ and gender‐based violence risks

When asked if participants would be interested in using HIV self‐test kits with their partners, potential risks of SGBV were raised. For instance a KI (HIV referral hospital) described:

Giving it to a sexual partner might bring violence because they may be like ‘why are you giving me this, you don’t trust me’. It can bring conflicts and questions, he or she might end up thinking you have other partners unless if you first give information to them. Not everybody can disclose his or her HIV status to a wife or husband at home.

Young women participants also described concerns about being coerced into testing by boyfriends, that in turn could lead to rejection if the result was positive: “Now the boyfriend can also give it to you but insist that you test in their presence and if you are HIV positive it can lead to separation” (FG women, 20 to 24 years, ID#9). Inequitable relationship power dynamics could result in harmful outcomes, leading some to highlight the need to work with men:

Some refugees when they come to us and a lady tests, we tell her, ‘Now you have tested, you are negative, why don't you bring your partner so that you test together?’ The lady will tell you that, ‘our men, if you tell them ‘let us test’, he will say that I am committing adultery, that I am having another man’. It would be easier for a man to convince a woman to test, than a woman convincing a man, because refugee women fear their husbands to tell them about testing. So some programs should also focus on the males, if we capture the males, women will just come along. (KI, humanitarian agency)

#### 3.3.3 Peer supported HIV self‐testing

When asked if they would prefer to receive an HIV self‐test kit from a peer educator, family member, friend or sex partner, most participants preferred peer educators. They regarded peers as trustworthy and reliable sources of knowledge. Others explained that a peer would also reduce stigma they may experience from families for being sexually active and may enhance confidentiality:

The peer educator because youth start sex very early, by fifteen they have a boyfriend they keep as a secret. So if you ask for it [HIV self‐test kit] from a family member they start wondering, because they know you were born HIV‐negative but now at sixteen years you are asking for a test kit, meaning there is something you are doing, you are no longer a virgin. I think a peer educator would be better. You cannot fully trust friends because sometimes you tell them something but again they tell others. (FG young women, 20 to 24 years, ID#7).

To sum up, two gender differences emerged across findings. First, although both young men and women discussed expectations of mistrust if they raised HIV self‐testing with partners, inequitable gender norms elevated women’s risks for SGBV, being left by male partners and being mistrusted if they test HIV positive. Second, participants spoke about preferences for peer educators in different ways. Young men preferred peer leaders for reasons of leadership, authority and trustworthiness, whereas young women discussed comfort, non‐judgement and trustworthiness.

**Figure 1:**
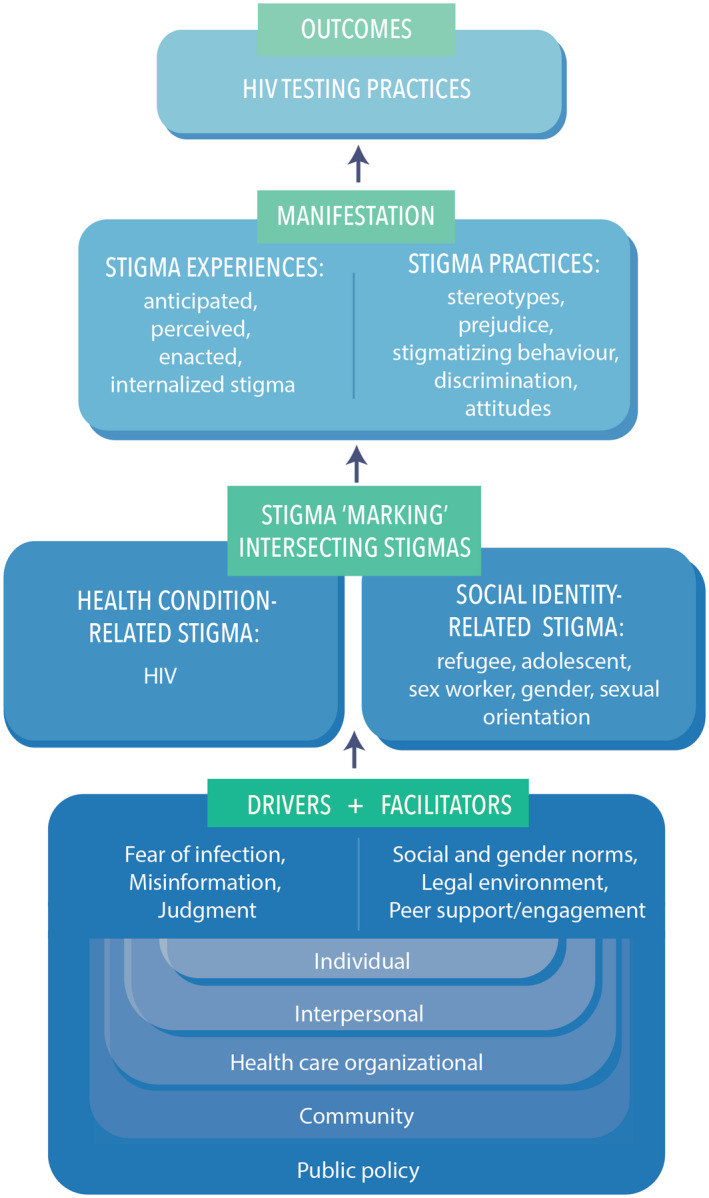
Applying the Health Stigma & Discrimination Framework [12] to understand intersecting stigma and HIV testing practices among urban refugee adolescents and youth in Kampala, Uganda.

We applied the HSD Framework [12] to contextualize findings in Figure 1. Fear of HIV infection, HIV misinformation and feelings of shame/blame exacerbated social inequities experienced by refugees, youth, sex workers, women, and people with HIV and together comprise *drivers of stigma* that reduce HIV testing. *Stigma facilitators* included legal precarity based on refugee status, sex work and same sex practices, as well as healthcare mistreatment and confidentiality concerns, which also reduce testing engagement. We provided examples of anticipated, enacted, perceived and internalized stigma that occurred across stigma drivers and facilitators. *Facilitators of enabling environments* [37] for HIV testing include self‐testing strategies and peer support. This framework highlights how these stigma experiences span social‐ecological levels, from laws, community norms, healthcare settings, interpersonal relationships and individual self‐perception.

## 4 DISCUSSION

Urban refugee youth described drivers and facilitators that produced intersecting stigmas that posed barriers to clinic‐based HIV testing while also increasing interest in HIV self‐testing. While HIV self‐testing, particularly peer‐delivered strategies, may mitigate stigma, underlying inequitable gender norms produced concerns that partner testing with this population may heighten SGBV.

Our study corroborates research on intersecting stigmas as a barrier to testing [13, 14] and signals the need for intersectional stigma reduction targeting HIV, adolescent, refugee and sex work stigmas. Findings underscore the salience of addressing stigma towards sexually active youth in community and healthcare spaces [11]. This aligns with quantitative findings that adolescent sexual and reproductive health (SRH)‐related stigma is linked with lower HIV testing among refugee young women [11]. Refugee young men’s negative experiences accessing clinic‐based HIV testing reflects research demonstrating higher adolescent SRH‐related stigma among young refugee men who accessed STI testing [38]. This suggests that adolescent SRH stigma may have multi‐directional effects as a barrier to, and outcome of, clinic‐based testing. It also signals the need for sex‐positive messaging for refugee youth’s SRH engagement. Young refugee sex workers identified similar HIV testing barriers as adult refugee sex workers in Kampala [39], and highlighted the need to address LGBT refugee young sex workers’ needs. Pervasive fear of HIV infection signals the need for tailored treatment literacy information.

High interest in HIV self‐testing among participants dovetails with evidence from youth across sub‐Saharan Africa [25, 26, 27] and adults in Uganda [28, 29]. HIV self‐testing is a strategy that responds to stigma, yet tailored implementation strategies are needed. Study findings document concerns over SGBV during partner testing, signalling that HIV services could be an entry point to addressing SGBV for refugee youth [40]. These concerns corroborate findings from adults in the Nakivale settlement that prioritize safety concerns over HIV testing [16]. Gender‐transformative approaches tailored for urban refugee youth can reduce SGBV and HIV risks [41, 42]. Finally, peer‐support as a preferred source of HIV self‐testing services aligns with research with adult refugees in Nakivale that social support can increase HIV testing [43]. Gender differences in preferences for peer support aligns with South African research that suggests that men may prefer male peers for perceived authority and beliefs that they will respect confidentiality [44].

Study limitations include the focus group design, whereby some participants may have felt uncomfortable sharing personal experiences. We only included cisgender women sex workers, missing the experiences of cisgender men and transgender sex workers. Given that participants were recruited from community partners, it is plausible they were more knowledgeable of HIV. Also, little is known of youth HIV testing experiences in refugee settlements so comparative research is warranted. Gender norms, roles and expectations could be more fully explored regarding HIV self‐testing. Despite these limitations, our study is unique in amplifying urban refugee youth HIV testing experiences and HIV self‐testing perspectives. Findings can inform tailored stigma informed HIV self‐testing interventions [12] (Figure 1).

## 5 CONCLUSIONS

Taken together, our findings signal the utility of the HSD Framework [12] for identifying the interplay between HIV, refugee and sex work stigma experienced across multiple life domains that limit HIV testing engagement with young urban refugees [45]. Findings can inform multi‐level strategies to foster enabling HIV self‐testing environments with urban refugee youth, including intersectional stigma reduction and leveraging peer support [37].

## COMPETING INTERESTS

We have no conflicts of interest.

## AUTHORS’ CONTRIBUTIONS

CHL was the principal investigator, conceptualized the study and manuscript, contributed to data analysis and led writing the manuscript. MO was a co‐investigator, contributed to study conceptualization and design, data acquisition, data interpretation and contributed to editing the manuscript. DKM contributed to study design, data acquisition and contributed to editing the manuscript. RH was a co‐investigator, contributed to study conceptualization and design, data acquisition and contributed to editing the manuscript. SM and PK were co‐investigators, contributed to study design and contributed to editing the manuscript. HA and LG contributed to data analysis and manuscript writing. JM contributed to editing the manuscript. LM and SB were co‐investigators, contributed to study design, manuscript writing and interpretation of results.
